# Human Prion Disease and Relative Risk Associated with Chronic Wasting Disease

**DOI:** 10.3201/eid1210.060019

**Published:** 2006-10

**Authors:** Samantha MaWhinney, W. John Pape, Jeri E. Forster, C. Alan Anderson, Patrick Bosque, Michael W. Miller

**Affiliations:** * University of Colorado at Denver and Health Sciences Center, Denver, Colorado, USA;; † Colorado Department of Public Health and Environment, Denver, Colorado, USA;; ‡ University of Colorado School of Medicine, Denver, Colorado, USA;; § Denver Veteran's Affairs Medical Center, Denver, Colorado, USA;; ¶ Denver Health Medical Center, Denver, Colorado, USA;; # Colorado Division of Wildlife, Fort Collins, Colorado, USA

**Keywords:** Creutzfeldt-Jakob syndrome, chronic wasting disease, prion diseases, transmissible spongiform encephalopathies, research

## Abstract

The transmission of the prion disease bovine spongiform encephalopathy (BSE) to humans raises concern about chronic wasting disease (CWD), a prion disease of deer and elk. In 7 Colorado counties with high CWD prevalence, 75% of state hunting licenses are issued locally, which suggests that residents consume most regionally harvested game. We used Colorado death certificate data from 1979 through 2001 to evaluate rates of death from the human prion disease Creutzfeldt-Jakob disease (CJD). The relative risk (RR) of CJD for CWD-endemic county residents was not significantly increased (RR 0.81, 95% confidence interval [CI] 0.40–1.63), and the rate of CJD did not increase over time (5-year RR 0.92, 95% CI 0.73–1.16). In Colorado, human prion disease resulting from CWD exposure is rare or nonexistent. However, given uncertainties about the incubation period, exposure, and clinical presentation, the possibility that the CWD agent might cause human disease cannot be eliminated.

## Abstract 2

Colorado death certificate data from 1979 through 2001 show that the risk for Creutzfeldt-Jakob disease did not increase for residents of counties where chronic wasting disease is endemic among deer and elk.

An emerging wildlife epizootic of chronic wasting disease (CWD) (*1*), a contagious prion disease among mule deer, white-tailed deer, and Rocky Mountain elk, has potential public health implications (*2**–**5*). CWD is related to other mammalian transmissible spongiform encephalopathies (TSEs), such as Creutzfeldt-Jakob disease (CJD) in humans, bovine spongiform encephalopathy (BSE) in cattle, and scrapie in sheep. In prion diseases, a normally produced cellular protein accumulates in an abnormal, misfolded, and aggregated form (*6*), which results in neuron destruction and a universally fatal outcome after a prolonged incubation period.

CWD infects wild and captive deer and elk in several US states and Canadian provinces. The highest reported disease prevalence is in a contiguous region, spanning parts of Colorado, Wyoming, and Nebraska (Figure 1), where the estimated disease prevalence is 5% in mule deer, 2% in white-tailed deer, and 0.5% in elk (*7*). CWD was first noted in captive deer at a research station in north-central Colorado near Fort Collins in the 1960s (*1*) and later in a wild elk near Estes Park in 1981 (*8*). No clear epidemiologic connections have been found between original cases and more recent cases, which suggests that unidentified risk factors may be contributing to the relatively wide and unpredictable geographic distribution of CWD (*2**–**4*).

**Figure 1:**
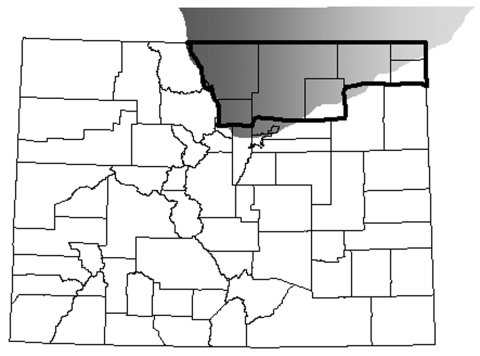
Location of chronic wasting disease (CWD)–endemic area in northeastern Colorado, USA (*7*) (gray shading) in relationship to Colorado counties regarded as CWD counties (bold outline) for purposes of comparing Creutzfeldt-Jakob disease rates and relative risk among resident human populations.

Humans and animals can acquire TSEs by consuming prion-contaminated food. Outbreaks of prion disease include an epidemic of kuru among the cannibalistic Fore tribe of the New Guinea highlands (*9*) and an epizootic of BSE in the United Kingdom, caused by feeding to cattle protein supplements derived from prion-infected cattle offal (*10*). Food-based prion transmission between species also occurs, although a phenomenon known as the "species barrier" decreases transmission efficiency. In vitro studies (*11**,**12*) indicate that this natural barrier reduces human susceptibility to animal prion diseases, including CWD. As yet, no cases of human prion disease have been linked with CWD (*5**,**13**–**15*), and natural transmission of CWD to humans or traditional domestic livestock seems unlikely (*2**,**3**,**5**,**12**,**14**,**16**,**17*).

The otherwise reassuring molecular evidence of species barriers is clouded by the disparate experiences with scrapie and BSE as foodborne human pathogens. Scrapie exposure has not been demonstrated to increase CJD risk, despite extensive human exposure (*18*). Conversely, in Britain the consumption of BSE-infected cattle led to an epidemic of variant CJD (vCJD), beginning in the mid-1990s (*19**–**23*). As of June 2006, however, only 161 cases of vCJD have been identified in the United Kingdom (*24*), despite the dietary exposure of millions of Britons to the BSE agent. In addition, recent studies indicate that large numbers of cases of vCJD are unlikely to occur in Britain in the future (*25*). Because the CWD agent is distinct from the BSE agent (*12**,**26**–**29*) and the type and degree of human exposure to these 2 agents differ, the risk for CWD transmission to humans cannot be directly extrapolated from the BSE and vCJD epidemics (*30*).

Because no completely reliable experimental animal model exists for testing the potential for CWD to cause CJD (*30*), human case investigations and epidemiologic studies remain valuable tools for assessing the potential risk associated with CWD exposure (*5*). Data that define human CWD exposure from consumption of infected deer or elk do not exist. However, in 7 northeastern Colorado counties (Boulder, Larimer, Logan, Morgan, Phillips, Sedgwick, and Weld) that are considered CWD-endemic areas (*7*) (Figure 1), the Colorado Division of Wildlife (CDOW) hunter license records indicate ≈75% (38,458 of 51,048) of deer and elk hunting licenses purchased from 1995 through 2001 were issued locally (CDOW, unpub. data), which suggests that county residents consume most regionally harvested game. Using Colorado death certificate data from 1979 through 2001, we modeled whether residence in a CWD-endemic county affected the risk-adjusted probability that a death is from CJD. We also examined whether the probability that a death is from CJD increased over time. To account for the possibility that CJD may have been misclassified, we also conducted sensitivity analyses using an expanded definition of event, similar to criteria used by Majeed et al. (*31*).

## Materials and Methods

### Study Population

Colorado death certificate data from 1979 through 2001 were used. Deaths during 1979-1998 and 1999-2001 were classified by the ICD-9 and ICD-10 codes, respectively. Sporadic CJD is extremely rare in persons <30 years of age (*32*), and vCJD cases have not been reported in patients <12 years (*33*). Therefore, we restricted all analyses to deaths occurring at >12 years, which provided 506,335 eligible deaths. We classified deaths as due to CJD if the codes 046.1 (ICD-9) or A81.0 (ICD-10) were listed as either the direct or contributory cause (events = 65).

**Figure 2:**
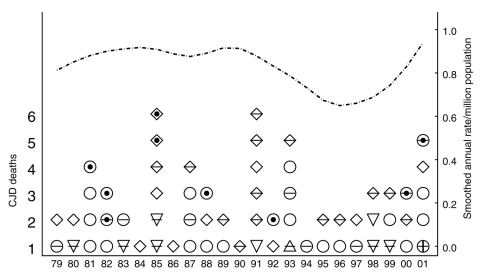
Colorado deaths 1979–2001 (left axis) with Creutzfeldt-Jakob disease (CJD) listed as the direct or contributory cause on the death certificate with age category at death identified by the plotting symbols 12–30 years (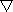), 31–55 years(∆), 56–70 years (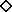) and >70 years (↓). Indicators of CWD-endemic county resident (black circle), female (–), and black (X) or other ( | ) race are also identified. On the basis of these death certificate data and Colorado demographic data (34), we also display a smoothed CJD death rate per million population over time (right axis) (∙ – ∙).

Additional Colorado death certificate data used included age at death, sex, and marital status. We considered marital status as a predictor, because it may influence whether symptoms are recognized, which subsequently increases the likelihood of diagnosing CJD. Years of education data were not collected before 1989; therefore, this variable was not considered as a predictor. Figure 2 contains individual characteristics for persons who died in Colorado with CJD listed on the death certificate and smoothed population CJD rates (*34*).

In 1998, the Colorado Department of Public Health and Environment (CDPHE) initiated human prion disease surveillance. From 1998 through 2001, CDPHE identified 20 Colorado resident deaths consistent with prion disease (Table 1). For 10 of these 20 deaths, CJD was confirmed by examination of brain tissue from biopsy or autopsy specimens. Three deaths were classified as probable CJD; rapidly progressive dementia clinically consistent with prion disease was supported by nonspecific tests. Seven of the 20 CJD deaths were classified as suspected CJD because the diagnosis was made without autopsy, biopsy, or supportive testing. In 14 of these 20 deaths, the ICD code indicated CJD. Inexplicably, the remaining 6 patients who died (4 with confirmed CJD, 0 with probable CJD, and 2 with suspected CJD) had a medical record consistent with CJD, but the deaths were not coded as such. Three of these deaths (2 with confirmed CJD, 0 with probable CJD, and 1 with suspected CJD) were identified under our expanded definition. CDPHE review of the death certificates for the 6 misclassified deaths found that CJD was not reported as a cause of death and that the ICD-10 codes were consistent with the stated cause of death.

**Table 1:** ICD codes and corresponding event classifications for human prion disease deaths of Colorado residents, 1998–2001*

Death y	CWD-endemic county	Sex	Death age, y	CJD status	ICD-9/ICD-10 codes†	Event classification
1998	No	F	>70	Probable	**046.1**	CJD/expanded
	Yes	M	>70	Confirmed	358.9, 507.0, 799.9	None‡
	No	F	31–55	Confirmed	**046.1**	CJD/expanded
	No	M	31–55	Confirmed	**046.1**	CJD/expanded
1999	No	F	31–55	Confirmed	**A81.0**	CJD/expanded
	No	M	56–70	Probable	**A81.0,** F17.1, I50.0, J44.9	CJD/expanded
	No	F	56–70	Confirmed	**G31.9**	Expanded
	No	F	>70	Suspected	**A81.0,** G20, **R29.8**	CJD/expanded
2000	No	M	56–70	Suspected	**A81.0**	CJD/expanded
	Yes	F	56–70	Confirmed	**A81.0,** E86, **G93.4**	CJD/expanded
	Yes	M	56–70	Confirmed	**G31.9,** J96.9	Expanded
	No	F	>70	Suspect	**A81.0,** J18.9	CJD/expanded
	Yes	F	56–70	Suspected	**G93.4,** F32.9, F41.9, J44.9	Expanded
2001	Yes	F	56–70	Confirmed	**A81.0,** I46.9	CJD/expanded
	No	M	56–70	Probable	**A81.0,** I48, I64	CJD/expanded
	No	F	56–70	Confirmed	**A81.0,** R53, R56.8	CJD/expanded
	No	M	>70	Suspected	**A81.0,** E87.8, **G96.9,** J18.9, N19, R99	CJD/expanded
	No	F	56–70	Confirmed	G20	None‡
	Yes	M	>70	Suspected	I10	None‡
	No	M	56–70	Suspected	A81.0, **I46.9**	CJD/expanded

*CWD, chronic wasting disease; CJD, Creutzfeldt-Jakob disease. †**Boldface** codes qualify for at least 1 event definition (Table A1 and Table A2). ‡Codes reported for cases missed on all definitions; 358.9, unspecified myoneural disorder; 507.0, pneumonitis due to solids or liquids, specifically due to inhalation of food or vomit; 799.9, asphyxia, other unknown and unspecified; G20, Parkinson's disease; I10, essential (primary) hypertension.

### Statistical Considerations

A review of Colorado death certificates identified 65 deaths with CJD listed on the death certificate from 1979 through 2001; from all causes, 81,916 and 424,419 persons >12 years died in the CWD-endemic and non–CWD-endemic counties, respectively. We were interested in testing whether the relative risk (RR) was greater than 1.0, where RR is the probability of a CJD death, given residence in a CWD-endemic county, divided by the corresponding probability in a non–CWD-endemic county. The RR is approximated by the odds ratio for a rare event such as death from CJD. Assuming a 2-sided χ^2^ test with a significance level of 0.05, we had >85% power to detect an unadjusted RR of 2.47. Assuming 65 CJD deaths, this corresponds to 21 (2.56 cases/10,000 deaths) and 44 (1.04 cases/10,000 deaths) deaths in the CWD-endemic and non–CWD-endemic counties, respectively.

We conducted separate analyses for the primary predictors of interest: residence in a CWD-endemic county and death year. The RRs and 95% confidence intervals (CIs) were estimated by using logistic regression in SAS (SAS Institute, Cary, NC, USA). Covariates in the multivariable analysis were death age, sex, ICD classification, and marital status. The CWD county analysis also was adjusted for death year. For death year, ICD classification was considered as an effect modifier.

## Results

### Characteristics of Persons Who Died

Descriptive characteristics by CWD endemicity of county are presented in Table 2. Due to the large sample size, statistical significance was observed for all covariates, although most differences were relatively small. Those who died in CWD-endemic counties were more likely to be white, >70 years of age, and married or widowed rather than divorced.

**Table 2:** Characteristics of persons who died at ages >12 years, Colorado, 1979–2001*

		CWD-endemic counties, N = 81,916 (16.18%); no. (%)	Non–CWD-endemic counties, N = 424,419 (83.82%), no. (%)	p value†
Age at death, y				<0.0001
	12–30	3,419 (4.17)	17,868 (4.21)	
	31–55	9,367 (11.44)	5,8379 (13.76)	
	56–70	16,182 (19.75)	94,684 (22.31)	
	>70	52,947 (64.64)	253,476 (59.72)	
	Unknown‡	1 (0.00)	12 (0.00)	
Education, y				<0.0001
	Unknown‡	31,788 (38.81)	167,480 (39.46)	
	<12	15,432 (18.84)	75613 (17.82)	
	12	16,843 (20.56)	95,337 (22.46)	
	13–16	13,853 (16.91)	70,115 (16.52)	
	>16	4,000 (4.88)	15,874 (3.74)	
Sex				<0.0001
	Female	40,665 (49.64)	204,864 (48.27)	
	Male	41,251 (50.36)	219,554 (51.73)	
	Unknown§		1 (0.00)	
ICD				0.0002
	1979–1998 (ICD-9)	68,479 (83.60)	356,978 (84.11)	
	1999–2001 (ICD-10)	13,437 (16.40)	67,441 (15.89)	
Marital status				<0.0001
	Single	6,701 (8.18)	40,806 (9.61)	
	Married	37,430 (45.69)	186,286 (43.89)	
	Divorced	7,419 (9.06)	48,062 (11.32)	
	Widowed	30,310 (37.00)	148,137 (34.90)	
	Unknown	56 (0.07)	1,128 (0.27)	
Race				<0.0001
	White	81,229 (99.16)	403,351 (95.04)	
	Black	213 (0.26)	16,243 (3.83)	
	Other	474 (0.58)	4,825 (1.14)	

*CWD, chronic wasting disease. †χ^2^ test. ‡Not recorded before 1989. §Excluded from χ^2^ test.

### Univariate Analyses

Univariate analyses allowed us to describe event characteristics. Table 3 contains the univariate RRs and corresponding 95% CIs for available predictors. CWD-endemic counties contributed 16.18% of total deaths but only 13.85% of deaths with CJD listed on the death certificate (p = 0.61) (Figure 2). This finding corresponds to an unadjusted CJD rate in CWD-endemic counties of 1.10/10,000 deaths; in non–CWD-endemic counties, this rate was 1.32/10,000 deaths. We saw a slight decrease in CJD risk over time (p = 0.54); 43.08% of CJD deaths occurred before 1989. CJD risk decreased with age of death; 46.15% of CJD deaths occurred in persons 56–70 years of age and 40.00% in those >70 years. Given this younger population, predictable changes occurred in the distribution of marital status.

**Table 3:** Univariate relative risk estimates of available risk factors for Creutzfeldt-Jakob disease, data from Colorado death certificates, 1979–2001*

Covariate		RR (95% CI), N = 506,335, events = 65
Age at death		p = 0.029
	Units = 10 y	0.87 (0.78–0.99)
CWD county†		p = 0.61
	No	1.0
	Yes	0.83 (0.41–1.68)
Death year†		p = 0.54
	Units = 5 y	0.95 (0.79–0.13)
Sex		p = 0.90
	Male	1.0
	Female	0.97 (0.60–1.58)
ICD-10		p = 0.83
	No	1.0
	Yes	1.07 (0.56–2.05)
Marital status		p = 0.0094
	Widowed	1.0
	Divorced	0.80 (0.23–2.85)
	Married	2.86 (1.51–5.42)
	Single	2.19 (0.86–5.57)
	Unknown	–‡
Race		p = 0.94
	White	1.0
	Nonwhite	2.87 (0.40–20.6)

*RR, relative risk; CI, confidence interval; CWD, chronic wasting disease. †Primary predictor . ‡Insufficient events.

### Multivariable Models

Table 4 contains the adjusted RRs for CWD endemicity of county and year of death. An RR >1.0 is consistent with the hypothesis of an increased risk for death from CJD, given residence in a CWD-endemic county. In the multivariable model, residing in a CWD-endemic county did not achieve statistical significance (RR 0.80, 95% CI 0.40–1.62). Death year remained not significant after adjusting for the additional covariates (for every 5-year increase, RR 0.92, 95% CI 0.73–1.16).

**Table 4:** Results for primary predictors from multivariable analyses for CJD and expanded event definitions, data from Colorado death certificates, 1979–2001*

Covariate		CJD, N = 506,335, events = 65 RR (95% CI)	Expanded age 12–55 y, N = 89,033, events = 339 RR (95% CI)	Expanded, N = 506,335, events = 1,911 RR (95% CI)
**CWD-endemic county**		**p = 0.55**	**p = 0.75**	**p = 0.48**
	**No**	**1.0**	**1.0**	**1.0**
	**Yes**	**0.81 (0.40, 1.63)**	**0.95 (0.69–1.31)**	**1.05 (0.93–1.18)**
**Death year**		**p = 0.48**	**p = 0.15**	**p < 0.0001**
	**Units = 5 y**	**0.92 (0.73–1.16)**	**0.93 (0.84–1.03)**	**0.81 (0.77–0.84)**

*CJD, Creutzfeldt-Jakob disease; RR, relative risk; CI, confidence interval; CWD, chronic wasting disease.

### Death Rates

**Figure 3:**
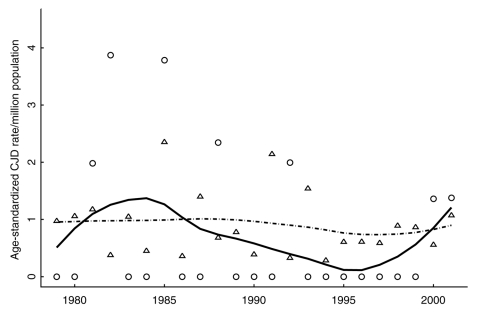
Annual age-standardized Creutzfeldt-Jakob (CJD) death rates per million population were calculated for chronic wasting disease (CWD)–endemic (ϒ) and non–CWD-endemic (∆) counties. Population rates were age-standardized to the 2001 age distribution for Colorado (*34*). We also display smoothed rates for the endemic (―) and non–CWD-endemic (∙ − ∙) counties.

In addition to analyzing death certificate data, we computed annual age-standardized CJD death rates per million population (Figure 3) for the CWD-endemic and non–CWD-endemic counties (*34*). These population rates were age-standardized by using the 2001 age distribution for Colorado. Smoothed age–standardized rates were similar to the crude population rates for Colorado shown in Figure 2 (smoothed median 0.88, range 0.65–0.94). As expected, given the smaller population size, more variability was observed for these rates in the CWD-endemic counties (smoothed median 0.67 per million, range 0.11–1.37), than the non–CWD-endemic counties (smoothed median 0.96 per million, range 0.73–1.01). Overall, annual crude population rates were slightly lower than age-standardized rates in both the disease-endemic counties (smoothed median 0.52 per million, range 0.09–1.29) and non–disease-endemic counties (smoothed median 0.85 per million, range 0.76–1.01) (data not shown).

### Expanded Definition Analyses

We considered that if CWD were transmissible to humans, then it might be manifested with different signs and symptoms than typical sporadic CJD, resulting in misdiagnosis or classification under a different ICD code. Therefore, in addition to assessing data for CJD, we conducted sensitivity analyses using an expanded definition (Table A1 and Table A2). This definition increased the number of event codes to 29 ICD-9 and 30 ICD-10 (events 1,911). These codes corresponded to neurodegenerative syndromes in which signs are exhibited that are prominent in some forms of prion disease. To minimize false-positive results, we did not consider death from Alzheimer disease after 55 years of age as an event. In the United Kingdom, most vCJD cases have occurred in persons <55 years, with a median age at death of 28 years (range 14–74 years) (*35*). In addition, among patients >55 years, the incidence of age-related neurodegenerative diseases tends to obscure all but dramatic increases in conditions that may be attributable to CWD exposure. Therefore, to increase specificity, we also considered the expanded definition restricted to deaths in persons 12–55 years, which provided 89,033 eligible deaths (events 339). The adjusted expanded definition RRs for CWD endemicity of county and year of death are contained in Table 4. Under the expanded definition, we see a decrease in risk over time (p<0.0001), although significance is lost when the analysis is restricted to deaths of those who died before the age of 55 years.

## Discussion

CWD has occurred in free-ranging deer and elk in northeastern Colorado for >25 years (*7**,**8*), so some persons likely have been exposed to the CWD agent. The human risk from exposure to CWD cannot be quantified because identifying exposed persons is not possible. The CDOW records indicate that ≈75% of deer and elk hunting licenses in 7 northeastern Colorado counties with high CWD prevalence are issued locally, which indicates that residents consume most game harvested in this region. Using Colorado death certification data from 1979 through 2001, we modeled the risk for a CJD death with CWD-endemic county residence as the exposure of interest. Similarly, we examined whether CJD deaths have increased overall. Given the possibility of misclassification of CJD and human TSEs, sensitivity analyses were conducted for expanded event definitions.

Human prion disease is rare, and increased risk due to CWD exposure appears to be subtle or nonexistent. No significant difference was found in the proportion of deaths from CJD in CWD-endemic versus non–CWD-endemic counties (adjusted RR 0.81, 95% CI 0.40–1.63). The upper CI value does not exclude an increased risk for CWD-endemic county residents, but it is inconsistent with a dramatic increase in that risk. Clearly, using residence in a CWD-endemic county as a surrogate for exposure has several limitations. The most obvious is that many persons with no history of hunting or deer and elk consumption are included in the exposed cohort. Conversely, exposed persons may live outside these counties. Given the potentially long incubation periods associated with prion diseases, ample opportunity would exist for infected persons to move from disease-endemic counties before the onset of illness. Moreover, other unrecognized risk factors (i.e., familial CJD or iatrogenic sources of infection) could confound epidemiologic investigations.

When Colorado CJD rates were examined over time, no significant change in CJD deaths was demonstrated (5-year RR 0.92, 95% CI 0.73–1.16). Although finding that risk for deaths from neurologic disease decreased over time under our expanded event definition is reassuring (5-year RR 0.81, 95% CI 0.77–0.84), this analysis should be interpreted with caution. The findings could be influenced by the lack of specificity in the definition and the switch from ICD-9 to ICD-10 codes in 1999. After excluding deaths in persons >55 years of age in the expanded definition, the results became inconclusive.

Although an increase in CJD deaths has not been observed in Colorado, due to the long incubation periods of prion diseases, infected persons may not have had sufficient time for disease to develop or may have left the state before disease onset. Although the prevalence and known range of CWD has increased over time (*2**–**4*), CWD exposure may be decreasing due to ongoing efforts by the public health and wildlife management agencies (*2**–**4*). Active education about CWD has been ongoing in northeastern Colorado since 1995. This information campaign includes several specific recommendations to minimize exposure for hunters, meat processors, and taxidermists (*4*). In addition, since 1994, testing has been available for game harvested in CWD-endemic counties, thereby removing a proportion of harvested, CWD-infected deer and elk from the human food chain.

Identifying cases of human prion disease remains a challenge. How human prion disease linked to CWD would be manifested clinically or pathologically is not clear. The probability of CJD being accurately diagnosed is influenced by changes in diagnostic practices; access to medical care, particularly specialized neurologic consultations; and the availability of diagnostic testing, including autopsy and postmortem pathologic examinations. Improved case ascertainment should result from the establishment of the National Prion Disease Pathology Surveillance Center, which offers free diagnostic testing, complemented by increased Colorado surveillance efforts, including classifying human prion diseases as a physician-reportable condition, funding to pay for autopsies, and outreach to neurologists, pathologists, and coroners (*36*). Increased publicity about BSE, CWD, and human TSEs may have led to changes in diagnostic practices or case recognition, particularly in CWD-endemic areas due to a perceived association of CWD with human disease.

Death certificate data undoubtedly underestimate the prevalence of CJD. A limitation of this study is that diagnosed human TSE cases may not be recorded as CJD on the death certificate. Between 1998 and 2001, CDPHE surveillance identified 6 persons who died with a medical history of CJD for whom CJD was not reported on the death certificate; therefore, those deaths were not captured as events in our survey, although 3 of these deaths were identified under our expanded definition. Given that CDPHE surveillance overlapped only the past 4 years of our study, we could not reclassify these additional TSE deaths as CJD without introducing an obvious bias in the analysis of year of death. As a post hoc sensitivity analysis to our primary CJD endpoint in the CWD county analysis, we reclassified these 6 missed cases as events and computed the unadjusted RR. Although including these cases changed the CWD county point estimate from 0.83 (95% CI 0.41–1.68) to 1.16 (95% CI 0.64–2.12), the results remained highly nonsignificant (p = 0.63). The results of this sensitivity analysis should be interpreted with caution as increasing awareness of CJD is unlikely to be uniform across a state or country. In our analysis, this heterogeneous distribution may have resulted in an increase in misclassification bias over time, such that reclassifying cases that were not identified on the death certificate led to identifying an excess of CJD that was unrelated to exposure in the CWD-endemic counties.

Despite increased scrutiny, evidence of increased CJD in Colorado has not yet been demonstrated. Smoothed Colorado CJD annual rates based on death certificate data are consistently <1 case per million population (median 0.88, range 0.65–0.94). In the United Kingdom, which arguably has the most comprehensive human prion disease surveillance, the annual crude mortality rates from sporadic CJD per million population were 0.86, 1.08, 0.84, and 0.57 in England, Wales, Scotland, and Northern Ireland, respectively, over the period from 1990 to 2003 (*35*). The overall mortality rate from sporadic CJD from 1999 through 2002 in Australia, Canada, United Kingdom, and 8 additional European countries was estimated to be 1.39 per million population >10 years, although rates were highly variable across countries (0.48–2.23) (*37*). Approximately 84% of Colorado's population is >10 years of age (*38*) such that the comparable median is 1.05 (range 0.77–1.12). Thus Colorado's CJD rates appear comparable to or below other reported rates.

Continued case surveillance remains crucial for identifying and characterizing human prion disease (*5*). Recognition of CWD transmission to humans will likely require the identification of a human TSE patient with a history of exposure to deer or elk, evaluation of the clinical course and pathologic features at autopsy, and characterization of the prion strain in laboratory studies. Additional epidemiologic studies, such as a case-control study or cohort study that compares hunter license data with death certificate data, also should be conducted. Until the health risks from CWD can be fully ascertained, preventative steps to reduce exposure to the CWD agent and other animal prion disease agents (e.g., BSE, scrapie) should continue (*5**,**30*).

CWD has existed in wild deer and elk of northeastern Colorado for well over 2 decades. However, neither the number of CJD deaths in CWD-endemic counties nor the rate of CJD in CWD-endemic counties or in Colorado as a whole have increased. Although our findings are consistent with those of other studies that suggest no connection between CWD and human TSEs (*5*,*12*), we cannot exclude the possibility that an isolated case of human disease associated with the CWD agent has occurred or may yet occur. However, our findings do suggest that death from CJD remains rare in Colorado.

## Appendix

**Table A1:** ICD-9 code classifications for expanded event definition, 1979–1998

Code definition	Age at death, y	ICD-9	Total with code* (12–55 y)†
Slow virus infection of the central nervous system	>12	046	0 (0)
Creutzfeldt-Jakob disease	>12	046.1	54 (8)
Other slow virus infection	>12	046.8	0 (0)
Unspecified slow virus infection	>12	046.9	0 (0)
Presenile dementia	12–55	290.1	8 (8)
Senile dementia, depressed or paranoid type	12–55	290.2	0 (0)
Cognitive or personality change of other type	>12	310.1	0 (0)
Other cerebral degenerations	>12	331	0 (0)
Alzheimer disease	12–55	331.0	18 (18)
Pick disease	>12	331.1	19 (3)
Senile degeneration of the brain	>12	331.2	55 (0)
Other cerebral degeneration	>12	331.8	13 (8)
Other cerebral degeneration, unspecified	>12	331.9	569 (35)
Other extrapyramidal disease and abnormal movement disorders	>12	333	0 (0)
Other degenerative diseases of the basal ganglia	>12	333.0	78 (8)
Myoclonus	>12	333.2	4 (1)
Other choreas	>12	333.5	11 (0)
Other and unspecified conditions	>12	333.9	22 (1)
Spinocerebellar disease	>12	334	0 (0)
Primary cerebellar degeneration	>12	334.2	7 (0)
Other cerebellar ataxia	>12	334.3	18 (1)
Cerebellar ataxia in diseases classified elsewhere	>12	334.4	0 (0)
Other spinocerebellar disease	>12	334.8	27 (6)
Unspecified spinocerebellar disease	>12	334.9	66 (6)
Encephalopathy, unspecified	>12	348.3	620 (183)
Sleep disturbances	>12	780.5	7 (1)
Abnormal involuntary movements	>12	781.0	22 (2)
Abnormality of gait	>12	781.2	13 (1)
Lack of coordination	>12	781.3	49 (0)

*Deaths may have more than 1 code reported. †Total deaths with code for persons ages 12–55 y.

**Table A2:** ICD-10 code classifications for expanded event definition, 1999–2001*

Code definition	Age at death, y	ICD-10	Total with code† (12–55 y)‡
Creutzfeldt-Jakob disease	>12	A81.0	11 (1)
Other atypical virus infection of CNS	>12	A81.8	0 (0)
Atypical virus infection of CNS, unspecified	>12	A81.9	0 (0)
Organic amnesic syndrome, not induced by alcohol and other psychoactive substances	>12	F04	0 (0)
Early-onset cerebellar ataxia	>12	G11.1	5 (1)
Late-onset cerebellar ataxia	>12	G11.2	0 (0)
Other specified degenerative diseases of basal ganglia	>12	G23.8	1 (0)
Degenerative disease of basal ganglia, unspecified	>12	G23.9	2 (0)
Myoclonus	>12	G25.3	2 (0)
Other specified extrapyramidal and movement disorders	>12	G25.8	2 (1)
Extrapyramidal and movement disorder, unspecified	>12	G25.9	5 (1)
Alzheimer disease with early onset	12–55	G30.0	0 (0)
Alzheimer disease with late onset	12–55	G30.1	0 (0)
Other Alzheimer disease	12–55	G30.8	0 (0)
Alzheimer disease, unspecified	12–55	G30.9	3 (3)
Pick disease	>12	G31.0	9 (0)
Senile degeneration of brain, not elsewhere classified	>12	G31.1	2 (0)
Other specified degenerative diseases of nervous system	>12	G31.8	3 (0)
Degenerative disease of nervous system, unspecified	>12	G31.9	56 (7)
Disorders of initiating and maintaining sleep [insomnias]	>12	G47.0	1 (0)
Sleep disorder, unspecified	>12	G47.9	1 (0)
Multisystem degeneration	>12	G90.3	15 (1)
Encephalopathy, unspecified	>12	G93.4	110 (34)
Disorder of central nervous system, unspecified	>12	G96.9	5 (1)
Other and unspecified abnormal involuntary movements	>12	R25.8	0 (0)
Ataxic gait	>12	R26.0	2 (0)
Other and unspecified abnormalities of gait and mobility	>12	R26.8	13 (0)
Ataxia, unspecified	>12	R27.0	9 (0)
Other and unspecified lack of coordination	>12	R27.8	0 (0)
Other and unspecified symptoms and signs involving cognitive functions and awareness	>12	R41.8	3 (1)

*CNS, central nervous system. †Deaths may have >1 code reported. ‡Total deaths with code ages 12–55 y.
